# Older persons’ perceptions about advanced directives and end of life issues in a geriatric care setting in Southwestern Nigeria

**DOI:** 10.11604/pamj.2019.32.64.17117

**Published:** 2019-02-05

**Authors:** Eniola Olubukola Cadmus, Lawrence Adekunle Adebusoye, Olufemi Oluwole Olowookere, Adebowale Taiwo Olusegun, Oluwagbemiga Oyinlola, Raimi Olalekan Adeleke, Olubukola Christianah Omobowale, Temitope Oluwagbenga Alonge

**Affiliations:** 1 Department of Community Medicine, College of Medicine, University of Ibadan, Nigeria; 2 Chief Tony Anenih Geriatric Centre, University College Hospital, Ibadan, Nigeria; 3 Department of Medical Social Works, University College Hospital, Ibadan, Nigeria; 4 Department of Orthopaedics and Trauma, University College Hospital, Ibadan, Nigeria

**Keywords:** Advanced directives, end-of-life decision making, older persons, Nigeria

## Abstract

**Introduction:**

Advanced directives enable the planning of care and support services independent of the older person’s ability to make the decision. There is a paucity of information regarding the views and preferences regarding advanced directives and other end of life issues among older persons in low and middle-income countries such as Nigeria. The study aimed to explore the knowledge, attitude and belief of older persons regarding decision making surrounding end of life and advance directives

**Methods:**

Data were collected through focus group discussions at a monthly social gathering of outpatients in a geriatric center in Oyo State, Nigeria. Discussions were audiotaped, transcribed and analyzed manually using a thematic approach.

**Results:**

Respondents’ knowledge about the end of life care and advanced directives as prescribed in high income settings were sparse and did not include choices about treatment options or any medical directives. The predominant perceptions among the participants bordered mainly on the arrangements for place of death, burial and property sharing. Participants listed in order of preference the major decision makers in the advanced directive process mainly, the oldest male child, religious leaders and legal practitioners.

**Conclusion:**

Our findings imply the need for improving knowledge and awareness about the benefits of advanced directives among older persons with a focus on opportunities for their active participation.

## Introduction

Death and dying is a reality that every individual must face at one point or the other in life. There is a high awareness of death among older persons by virtue of the fact that they have experienced death among a loved one and are themselves managing at least one morbidity [1]. Due to population aging many older persons are exceeding previous life expectancy and are expected to live more years in good health [2]. However, for most elderly people there is a constant reminder that life lived is approaching its end. Therefore, there is a need to make adequate provisions for the eventuality of death. There is a plethora of research about living healthy lives and successful aging [3-6]. However, there is a paucity of information on end of life issues including advanced directives among the older persons in many low and middle-income countries (LMIC) including Nigeria. Advanced directives are “documented instructions by a patient to ensure their medical preferences are fulfilled in the event that they can no longer communicate with their clinicians or family members” [7]. Further, end of life decision making also include treatment options or preferences before death, designation of a power of attorney for health care and decisions regarding a living will [8]. The concept has risen to consciousness particularly in high-income countries (HIC) especially in the wake of longer life expectancy and increased life-sustaining technology and therapy [9]. Research, however, shows that advanced directives, when properly utilized, is associated with benefits including the promotion of patient autonomy and quality of life [7].

Previous research on advanced directives have focused on the needs of patients with terminal illness [10, 11]. In addition, research has shown that caregivers including physicians and family have their preferences regarding what patients need at the terminal stages of life and provisions made after death [11]. However, the views of older persons regarding the end of life issues are needed to assist in planning support services for them during this vulnerable period of their life. Furthermore, evidence from HIC suggests that patient’s personal values have a huge impact on the dignity of death and dying [9]. However, in the traditional African setting, dying and death have different connotations compared to the high-income countries [12, 13]. Patient involvement in decision making has been shown to increase the quality of life at the end of life [11]. End of life decision making in previous studies have centered on the ability to die a peaceful death surrounded by loved ones, preferably at home [9, 13]. However, research shows that there are social predilections when it comes to decision making for advanced care planning and end of life decisions [8]. For instance, Hopp and Duffy (2003) in their study showed that race was a significant predictor of advanced care planning and treatment decisions [8]. The authors revealed that compared with whites, blacks were less likely to discuss end of life care with their family members. However, Waite *et al.* (2013) reported other factors shown to influence planning and decision making. These include, sociodemographic characteristics such as age, gender, educational level, marital status and availability of children [7]. No previous study has documented preferences of older persons regarding advanced directives and end of life (EOL) decision making in LMIC such as Nigeria. This study explored how out-patients, registered at a specialist geriatric center perceived issues around death, dying and end of life decision making. Information thereby obtained will assist in strengthening the knowledge base for end of life care and equip with health care providers with a better understanding of ways to provide adequate care and support for older persons in this stage of the life course.

## Methods

### Study design

This study was conducted at the Chief Tony Anenih Geriatric Centre (CTAGC), University College Hospital (UCH), Ibadan. The center provides specialized care for the elderly in Oyo State, south-western Nigeria. Using a qualitative approach, a total of five Focus Group Discussions (FGDs) were conducted over a one-month period in November 2016. Older males and females attending the Elders forum, a monthly social gathering of clients registered at the Centre. This forum serves as an opportunity for healthy older persons to mix and interact with their contemporaries outside their homes and is usually highly subscribed. For the purpose of this study, persons aged 60 years and above at the time of the study were considered eligible to participate. Older persons, who could not provide information themselves due to dementia, dumbness, deafness, psychiatric illness or any other communication problems, were excluded from the study. The participants consisted of low- and middle-class inhabitants according to Nigerian socio-economic classes [14]. The main languages spoken in the areas were English and Yoruba and were used for the interviews. For this study, advanced directives is defined as ‘a process of giving advance directive on what to do when individuals are not conscious to make informed decision about his will or how to go about his treatment [13]. Specifically, study participants were asked if they had ever heard of the term ‘advanced directives’ and to list what it entails as well as the importance of such instructions. In addition, views of the respondents concerning their preferred choice to make important decisions when they were no longer able to do so were explored.

### Data collection

The group discussions were used to explore the views of a purposive selection of older persons concerning death, dying advanced directives and other end of life decision making. Each focus group was made up of 6-8 people, a moderator and a recorder and was conducted in either English language, or the local language, Yoruba. Informed verbal consent was obtained and participants were assured of strict confidentiality and anonymity of data collected. Furthermore, attempts were made to ensure that the atmosphere was conducive for the discussion. The discussions were facilitated by a female and two males i.e. the first, fourth and fifth authors respectively. All facilitators were bilingual. The first author had training in qualitative research methods while the other two had a background in medical sociology and health communication. A semi-structured interview guide comprising open-ended questions and suitable probes was designed collectively by the research team members and was used for all the discussions. The guide was translated to the local language (Yoruba) for ease of communication and subsequently back-translated to English to ensure the original meanings were maintained before administration. The FGD topics covered included: common beliefs about illness/diseases among the elderly, views and cultural belief of older persons regarding death, dying, advanced directives and other end of life issues. The discussions were audio-recorded while a designated note taker took detailed notes to assist in the transcription process. The first and fifth authors took turns in supervising all the discussions and acted as observers when they were not facilitating a discussion. Each discussion lasted for about 60-90 minutes. At the end of each FGD, there was a debriefing between the moderator, scribe and the authors to discuss the emerging themes and possible differences from the other FGDs. After five FGDs had been conducted, we felt that saturation had been achieved and no new information could be obtained. All interviews were audio-recorded and transcribed verbatim.

### Data analysis

All transcripts were read several times by the authors and emerging themes were coded. Themes were identified by the researchers who initially worked independently, then subsequently in consort, using a qualitative thematic analysis approach. Each reader independently reviewed the transcripts and emerging themes were agreed upon. Where there was disagreement on the categorization of any theme, the authors had a discussion which enabled them to come to an acceptable conclusion. Attempts to maintain trustworthiness was achieved by giving two authors (EOC and OO) who had competence in qualitative data analysis the transcripts and codes hitherto generated.

### Ethical consideration

Approval to conduct the study was obtained from the University of Ibadan/University College Hospital Ethical Review Committee. Participants were informed of their right to decline or withdraw from the study at any time without any adverse consequences. A pamphlet introducing the researcher and describing the nature and procedures of the study, as well as the personal benefits, risks and rights related to participating, was read to all the participants. Verbal consent was obtained from each participant and a commitment was made by the investigators to safeguard the confidentiality and anonymity of the data collected.

## Results

In this study, we explored the participant’s views about good health, beliefs about illness and dying qualities of a dying person and cultural beliefs surrounding death and dying. We also analyzed the participant’s perceptions of advanced directives and the decision process. A total of 34 older persons participated in the eight focus groups with a mean age of 70.9 years (range 60 to 92 years). Participants provided a range of insights about death, dying and instructions for advanced directives. Majority of the participants were from the Yoruba tribe.

### Respondents’ views about health, access to health services and observed challenges

Health was described in terms of physical, mental and social well-being and as an integral part of successful aging and normal functioning.

*“Health is wealth… being healthy is having everything” (Male Participant FGD 1)*

Poor health, was reported to include mobility issues and poor agility due to diminished strength as a result of old age. Participants were of the opinion that illness goes beyond physical issues but may be as a result of other circumstances such as inadequate care, poor family support as well as being childless. Poor health, as reflected by the participant strips the individual of independence and autonomy. Most of the respondents considered unhampered mobility, functional independence and good mental status as important contributors to good health. The health of the family was also a considered advantage and contributed to social relations as well as a feeling of happiness.

In terms of access to health services, participants opined that these were not accessible to them due to an inability to afford payment. Other, barriers to health access were described and these included, long distances to available facilities as well as poor transport provision. Reasons given for the present situation included poor political will of the government in providing dedicated support and services for the elderly. Also, the economic downturn and high unemployment level among the youth in the country which limits the ability of the older persons and their main careers to finance their health care were also identified as barriers to health access.

### Views about dying and qualities of dying elderly persons

Most of the respondents were of the opinion that death is inevitable and may be sequelae of illness and unhappiness. Different qualities of a dying person were identified. For instance, symptoms of a dying individual include lack of communication, inability to perform normal activities of daily living as well as sensory issues such as perceiving strange smells or having visions. This view was reflected by many of the respondents.

**“You know an individual is dying when such an individual starts smelling the grave and they start seeing the life beyond” (Female participant FGD3)**

Some of the respondents opined that death was as a result of karma and may be peaceful or otherwise based on the individual’s behavior while on earth. In addition, there were other hereditary and faith-related beliefs surrounding death. For instance, some of the participants proposed that illness may occur as a result of a spiritual attack or may be a form of punishment for previous bad behavior.

*Advanced directives, issues related to death and dying and afterlife*

Regarding knowledge about advanced directives, most of the participants claimed they were aware of the term. However, when asked about what constituted advanced directives, none of the responses included their choices about health care and life sustaining medical treatment. The most common examples of advanced directives given were with regards to the choice of the burial site, instruction about body preservation, burial ceremony and property sharing. Furthermore, a large proportion of the respondents were of the opinion that advanced directives also included provisions for making a will. Most of the older persons thought that their children, especially the first male son, were the best persons to make the final decision. However other highly rated decision makers include religious leaders (pastors and Islamic clerics) as well as legal practitioners. When asked about the relevance and importance of the advanced directives, almost all the respondents highlighted the importance of leaving some form of instructions to guide their relatives upon their demise. This is because such laid down directives will guard against conflicts and unnecessary influence from persons deemed outside the family. For instance, one patient said:

**“I have my will written out, who I want to be invited to the funeral. I have my obituary. That gives me a sense of completion that I don´t have to put that burden on someone else. It´s to prepare myself for it.” (Male participant FGD3)**

Identified barriers to implementation of advanced directives include the cost of legal fees as well as family dynamics including disharmony and polygamy as well as traditional rites and practices laid down by the culture. In addition, some of the participants were of the opinion that due to poverty and the desire for property grabbing, laid down guidelines may not be followed through by family members. Regarding the choice of place of death, most of the participants preferred the home or familiar setting surrounded by loved ones including spouse and children. Quite a number of the participants expressed their desires for a quick burial thereby avoiding preservation and post-mortem.

## Discussion

This study set out to describe views about death, dying, advanced care planning and end of life issues among patients attending a geriatric center. Majority of the discussants in our study had a view that death is inevitable and unavoidable; as everyone will at a point, die. This is similar to a finding from studies conducted on issues related to death and dying, where old people accepted death as inevitable and almost all were happy with how well they had lived their lives [9, 13]. Likewise, in other studies, death was described as inevitable and a debt that everybody has to pay, sooner or later [13, 15]. From this study, discussants perceived that when some distinct traits are observed in an old person, the person is said to be dying. One of these traits is lack of effective communication. The participants observed that when an older person can no longer effectively communicate or interact with people around him, then such person is dying. This finding is similar to what was reported by Nguyen KT (2012) [10]. Furthermore, participants in this study reported that having funeral arrangements made, helping others, coming to peace with God and not being a burden were a good preparation for death [13, 16].

However, in this study, participants did not dwell on treatment preferences before death. This finding is quite different from what is obtained in HIC. A possible reason for this difference may be due to the fact that as expected, in the traditional African setting, older persons in this study considered death inevitable and out of their purview. Likewise, a previous study by Hopp and Duffy, (2003) revealed racial differences and showed that Blacks were less likely to engage in advanced care planning and to limit care at the time of death whereas their white counterparts were more likely to subscribe to limit care in certain situations and subscribed to withholding treatment [8]. As such, preparations for death and end of life decision making in this study focused mainly on selection of the individual responsible for major decision making, setting up a will and planning the preferred place of death. Other decisions mentioned included making provisions about burial rites and other funeral arrangements. These findings were similar to report in a high-income setting by Steinhauser *et al.* [16]. Some of the participants in our study were of the opinion that decisions after death such as property sharing, choice of burial sites should be left to their children, mostly the first son and probably the spiritual clerics. This is related findings from other studies where older persons preselected individuals whom they preferred to make decisions on their behalf after death [13, 16].

From this study, most discussants preferred to die in their homes or familiar settings. A similar study conducted in Ibadan also revealed that, older persons eminently preferred to die at home [13]. Likewise, findings from HIC revealed that older persons desired to die in their home although only a small percentage had the opportunity to do so [17, 18]. Our study also revealed that many of the older persons would prefer avoidance of post-mortem and quick burial within few days of death. This is in line with findings from a similar study among older persons in Oyo State where the older persons were reported to warn their families against taking their dead bodies to the mortuary [13]. The family has a huge role to play in decision making and end of life communication and preferences [1, 11]. Research shows that older persons prevail on the importance of naming someone to make decisions regarding their end of life care [16]. In our study, top preferences were male children and religious clerics. Death and dying surrounded by the family members were deemed important by the participants and similar findings have been reported in high-income settings as well [16, 19]. Also, efforts to be at peace with the supreme being/maker was frequently mentioned and is not uncommon in the traditional African setting [13]. Barriers to effecting the advanced directives in this study were identified as the high legal fees to prepare a will, the structure of the family particularly polygamy, cultural practices and traditional rites.

### Limitation

This study is not without limitations. First, the participants were chosen among those who attended a social gathering in the hospital setting and may have led to some selection bias as these individuals have potentially good health and health-seeking behavior. Furthermore, information to explore deeper necessary levels of preparation to influence the provision of medical care was not obtained.

## Conclusion

This study explored the attitude and perceptions of older persons attending a geriatric center in Oyo State, southwestern Nigeria and provides additional knowledge from the views of older persons themselves regarding advanced directives and end of life decision making. There was scarcely any mention of medical/physical intervention to prolong life, most of the directives centred around arrangement for place of death, burial and property sharing and corroborates findings from similar studies among people of African race and blacks in general. The study findings contribute to knowledge surrounding successful death and dying in the traditional African setting. Information hereby obtained will assist in the provision of patient-centred care with a view to improving the overall quality of life of the elderly towards the end of life.

### What is known about this topic

- There is a high awareness of advanced directives and end of life decision making in high income countries;
- Advanced directives promote patient autonomy and improves quality of life;
- Hitherto, the focus and recommendations for advanced directives were among terminally ill patients.

### What this study adds

- The views of older persons regarding the end of life decision making in a typical low income setting focused mainly on place of death, body preservation and funeral arrangement;
- Most older persons preferred to have their children (first male son) as the major decision makers after their demise;
- Barriers to implementation of laid down directives were mostly high legal fees and cultural rites and practices.

## Competing interests

The authors declare no competing interests.
